# Case report: Benign porta hepatic schwannoma

**DOI:** 10.4103/0971-3026.54888

**Published:** 2009-08

**Authors:** Naveen Kulkarni, Sajan J Andrews, VRK Rao, KV Rajagopal

**Affiliations:** Department of Radiology, Kasturba Medical College, Manipal–576 104, Karnataka, India

**Keywords:** Obstructive jaundice, porta hepatis, Schwannoma

## Abstract

Schwannoma is a myelin sheath tumor that can occur almost anywhere in the body. The most common locations are the central nervous system, extremities, neck, mediastinum and retroperitoneum. Benign schwannomas in the porta hepatis are extremely rare and radiologically are diagnosed as either enlarged lymph nodes or bowel masses, such as gastrointestinal stromal tumors. In this location they usually produce symptoms by compressing adjacent structures and often present with obstructive jaundice. The preoperative diagnosis can be extremely difficult.

## Introduction

Benign schwannomas, also referred to as neurilemomas and neurinomas, are encapsulated myelin sheath tumors. They are usually benign and their presentation depends on the anatomical location and tumor extent.[1] A benign schwannoma in the porta hepatis may be completely asymptomatic, being detected incidentally,[1,2] or may present with features of obstructive jaundice. In the literature, only two cases of benign schwannoma in the porta hepatis have been reported till date.

## Case Report

**Figure 1 (A, B):**
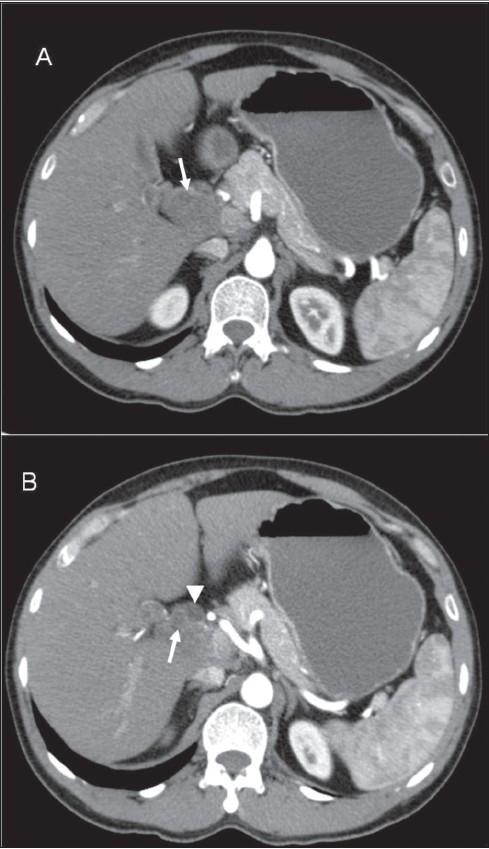
Axial contrast-enhanced CT scans show a heterogeneous soft-tissue lesion (arrow) in the porta hepatis with a dilated common bile duct (arrow head)

**Figure 2:**
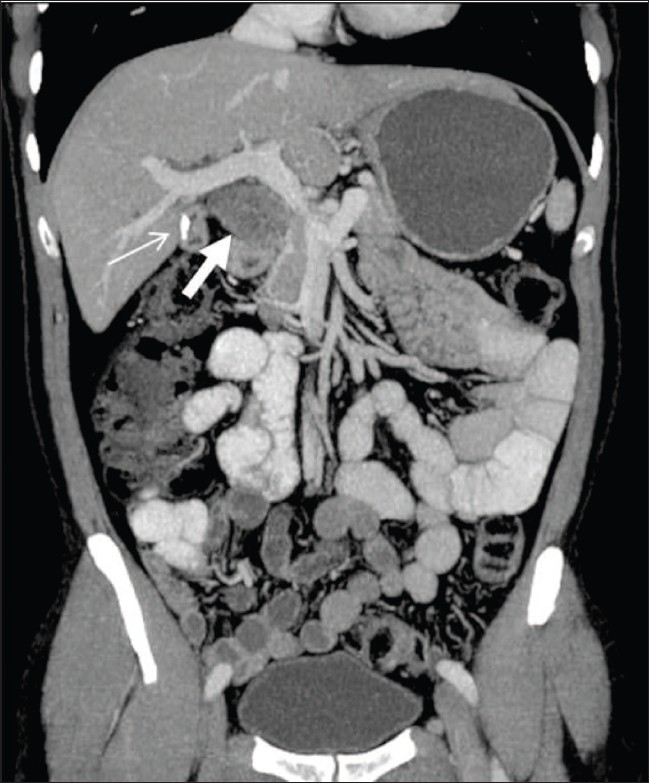
Coronal reformatted CT scan reveals a lesion (thick white arrow) in the porta hepatis extending caudally till the level of the duodenum with dilated intrahepatic biliary radicles. Also seen is a gallstone within a partially distended gallbladder (thin white arrow)

**Figure 3 (A, B):**
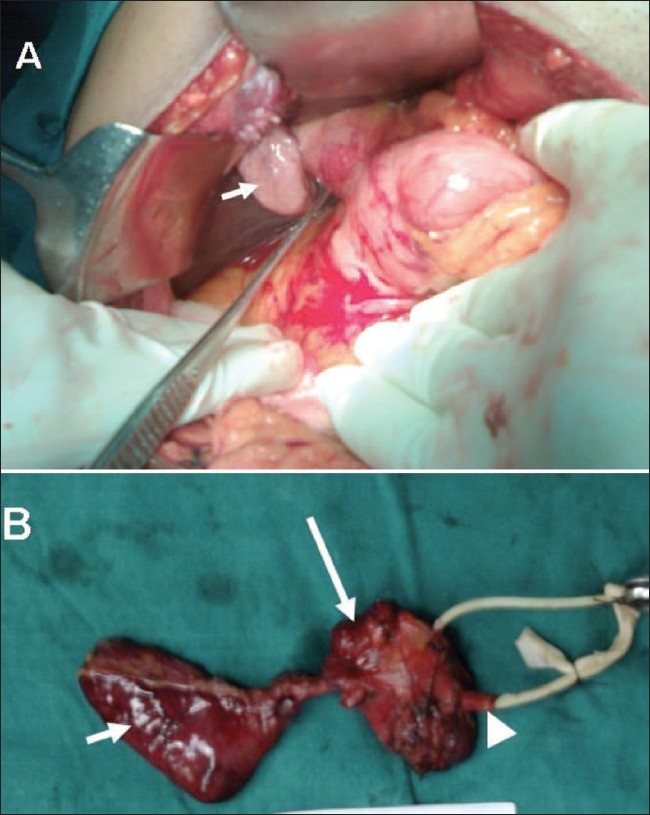
Intraoperative view of the lesion (A) with a collapsed gallbladder (short white arrow). Gross inspection (B) shows the resected tumor (long white arrow) involving the common bile duct (arrowhead) along with the gallbladder (short white arrow). The patency of the common bile duct is evident by the wire in-situ within the lumen of the common bile duct (arrowhead)

**Figure 4:**
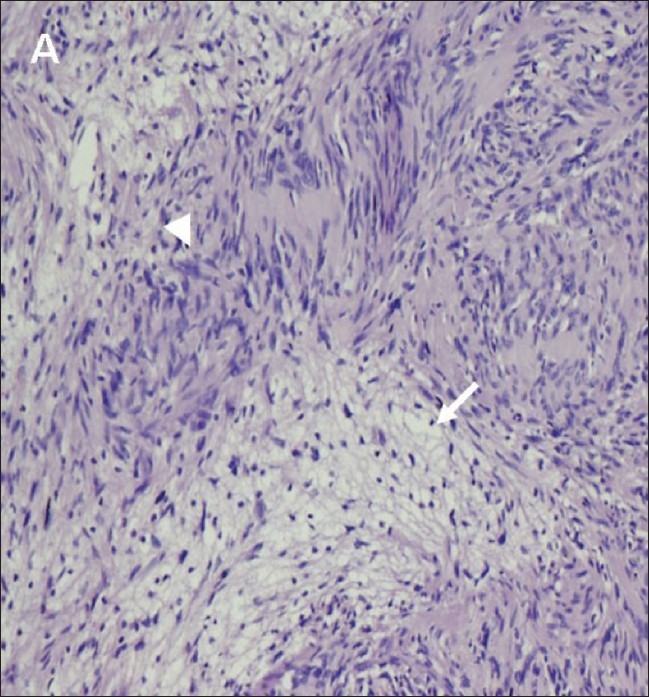
Photomicrograph (original magnification, ×200; hematoxylineosin stain) reveals that the tumor consists of focal hypercellular (Antony A) areas (arrowhead) and hypocellular (Antony B) areas (short arrow). The tumor cells are separated by loose stroma

A 38-year-old man was admitted to our institute with upper abdominal pain, poor appetite, loss of weight and yellowish discoloration of the eyes for 2 months. His clinical and family histories were noncontributory. There was no history of drug intake. Per abdominal examination revealed a soft abdomen with no evidence of hepatomegaly or splenomegaly. There was no lymphadenopathy. Laboratory investigations revealed elevated values of total bilirubin (7.4 mg/dl), direct bilirubin (4.3 mg/dl) and alkaline phosphatase (567 U/l). Total protein, albumin, ALT (alanine aminotransferase) and AST (aspartate aminotransferase) were normal. These findings were consistent with obstructive jaundice. CT scan of the abdomen revealed a heterogeneously enhancing lesion measuring 4.7 × 3.4 × 3.2 cm (craniocaudal × anteroposterior × transverse) in the region of the porta hepatis [Figures 1, 2], extending caudally till the second part of the duodenum. The lesion was seen to be compressing the supraduodenal portion of the common bile duct, causing dilatation of the proximal common bile duct and the intrahepatic biliary system. The patient underwent exploratory laparotomy, which revealed a 5 × 4 cm firm lesion [Figure 3A] that was inseparable from the common bile duct. Cholecystectomy and Roux-en-Y hepaticojejunostomy were performed and the lesion, along with the common bile duct, was excised. The resected specimen [Figure 3B] showed the lesion inseparably attached to the common bile duct and the gallbladder. The histopathologic findings were consistent with schwannoma composed of Antoni A and B areas [Figures 4].

## Discussion

Benign schwannoma is a tumor arising from Schwann cells, which form the neural sheaths of the peripheral nerves. The most common locations are the cranial nerves (especially the eighth cranial nerve) and the peripheral nerves in the region of the neck, mediastinum and extremities.[3] Theoretically, the tumor can affect almost any organ or nerve trunk in the body other than the optic and olfactory nerves, which do not possess Schwann cells.[3] Our review of literature showed that two cases of benign schwannoma in the porta hepatis and nine cases of extrahepatic biliary schwannoma have been reported in the literature.[4] Patients with schwannoma in the porta hepatis may be asymptomatic or may present with obstructive jaundice.[5,6] Gastrointestinal schwannomas appear as sharply demarcated, round or oval mass with homogeneous attenuation on both unenhanced and contrast-enhanced CT scans. They frequently lack a tumor capsule, necrosis, cavitation and calcification on CT scan.[7]

In the present case, CT scan revealed a heterogeneous lesion at the porta hepatis, which was initially suspected to be a lymph nodal mass or an exophytic gastrointestinal stromal tumor (GIST). Gastrointestinal stromal tumors are a distinct group of mesenchymal tumors arising from the muscularis propria layer of the digestive tract; they often project exophytically and/or intraluminally.[8,9] On CT scan, GISTs are usually well-demarcated spherical masses, with evidence of necrosis and cavitation within the lesion, both of which are uncommon in gastrointestinal schwannomas.[7,10] The other likely differential diagnosis of a solitary lesion occurring at this site is a polypoid extrahepatic cholangiocarcinoma.[11] A clinical diagnosis of schwannoma or polypoid extrahepatic cholangiocarcinoma is difficult unless the mass is palpable.[12]

On histopathology, the characteristic features include the presence of alternating Antoni A and Antoni B areas. The Antoni A area is composed of spindle-shaped Schwann cells arranged in interlacing fascicles. The Antoni B area consists of a loose meshwork of gelatinous and microcystic tissue. Large, irregularly spaced, thick-walled blood vessels can be seen in Antoni B areas. These may contain intraluminal thrombi.[13]

This case illustrates the difficulty in making the diagnosis of a schwannoma in the porta hepatis based on CT imaging features alone. Schwannoma can be mistaken for GIST and polypoid extrahepatic cholangiocarcinoma, and it is not possible to pre-operatively make a diagnosis based on imaging findings alone.[14]

In this case, the postoperative course was uneventful and at a follow-up visit after 3 months, the patient was clinically asymptomatic and free of icterus.
